# Body Weight Changes and Associated Social Factors in Newly Recruited Students at a Mexican Public University

**DOI:** 10.1002/osp4.70102

**Published:** 2026-04-25

**Authors:** Wendy Paola Gastélum Espinoza, Alma Marlene Guadrón Llanos, Gisela Duarte de la Peña, Francisco Castro Apodaca, Loranda Calderón Zamora, Dalia Magaña Ordorica, Yuridia Chaidez Fernández, Javier Abednego Magaña Gómez

**Affiliations:** ^1^ Programa de Nutrición Campus Culiacán Universidad Autónoma de Occidente Culiacán Sinaloa Mexico; ^2^ Laboratorio de Diabetes y Comorbilidades, Posgrado en Ciencias en Biomedicina Molecular, Facultad de Medicina Universidad Autónoma de Sinaloa Culiacán Sinaloa Mexico; ^3^ Facultad de Ciencias de la Nutrición y Gastronomía Universidad Autónoma de Sinaloa Hospital Civil de Guadalajara Fray Antonio Alcade Culiacán Mexico; ^4^ Posgrado en Ciencias en Biomedicina Molecular, Facultad de Medicina Universidad Autónoma de Sinaloa Culiacán Sinaloa Mexico; ^5^ Facultad de Ciencias de la Nutrición y Gastronomía Universidad Autónoma de Sinaloa Culiacán Sinaloa Mexico

**Keywords:** adolescent behavior, lifestyle, obesity, weight gain

## Abstract

**Introduction:**

The transition from high school to college involves lifestyle changes that can lead to increased body weight, a phenomenon commonly referred to as the Freshman 15. Although educational settings may differ across countries, this period could also present a risk of weight gain.

**Objective:**

To determine the weight gain in Mexican students during the transition from high school to a public university and identify the primary associated modifiable factors.

**Methods:**

Two hundred twenty‐six students of both sexes, aged 17–21 years, were evaluated at the beginning and end of the first semester. Anthropometric characteristics, lifestyle habits, and self‐perceived stress were analyzed. Intra‐subject differences were assessed using the general linear model with repeated measures, and categorical variables were evaluated using non‐parametric tests.

**Results:**

A significant weight increase of 0.8 kg was observed regardless of sex. Adolescents who ate under 20 min or lived in a rooming house without appliances exhibited the highest weight gain. Regression analyses revealed that female sex (*β* = −0.929, *p* = 0.008) and eating time of 21 min or more (*β* = 0.756, *p* = 0.050) were significantly associated with body weight change. In the multiple regression model, only sex remained a significant factor (*p* = 0.018).

**Conclusions:**

Weight gain in university students, especially in men, highlights this stage as a vulnerable period. It is essential to study habits and behaviors according to gender to design interventions that promote healthy eating. Further research is needed to identify modifiable factors associated with weight gain.

## 1 Introduction

Obesity represents a major global public health challenge, with a steadily increasing prevalence that impacts individuals across all age groups and regions. Approximately 2.5 billion adults are overweight, with over 650 million classified as obese. In 2024, 35 million children under 5 years of age and over 390 million children and adolescents aged 5 to 19 had overweight or obesity [1]. In Mexico, the prevalence of overweight and obesity among adolescents aged 12 to 19 years increased from 34.9% in 2012 to 43.8% in 2020. Among adults, over 70% have been affected by overweight and obesity in the past decade, indicating a growing trend [2].

The transition from high school to college or university is a critical and vulnerable period marked by accelerated weight and fat gain, as well as the adoption of unhealthy lifestyles, which can have long‐lasting effects into adulthood [3, 4, 5]. In Canada and the United States, there is a widespread belief among adolescents that students gain approximately 15 lbs (7 kg) during their first year of college. This phenomenon is known as the Freshman 15 [6].

Investigations have not consistently found significant changes in body weight. Some studies report weight gain ranging from 0.5 to 13 kg. These variations have been associated with a combination of lifestyle, personal, and environmental factors, including decreased physical activity, poor‐quality diet, impaired sleep hours, high levels of perceived stress, living independently, lower eating self‐regulatory skills, roommate weight, lack of time and motivation to eat healthily or engage in exercise, and the perceived limited availability of healthy food options [7, 8, 9, 10, 11]. Another consistently reported predictor of weight gain during the freshman year of college is weight suppression, defined as the difference between the maximum weight in adulthood (not due to medical reasons) and the current weight [12]. Although weight suppression and dietary restraint are often considered clinically relevant risk factors for the development of disordered eating, empirical evidence supporting a direct causal relationship remains limited and inconsistent [13]. For example, studies in female college students have shown that while modest weight gain during the freshman year is not directly associated with dietary restraint, disordered eating behaviors are more likely to emerge in individuals reporting higher levels of restraint and concerns about weight gain [14, 15]. These findings suggest that dietary restraint may be more strongly related to vulnerability to disordered eating than to weight change itself [16]. Therefore, interpretations regarding the relationship between weight loss and eating disorders should be made with caution, acknowledging the multifactorial nature of these associations and the need for further longitudinal and mechanistic research.

While much of the evidence on weight change during the transition to college has been generated in North American settings, these factors may manifest differently in other cultural contexts. In Mexico, universities generally do not provide student accommodation, so it is common for students to leave their parental homes and rent a house or room. This living arrangement can influence lifestyle behaviors and contribute to weight gain. While studies in Mexico have primarily examined the Freshman period from the perspective of depression, educational outcome, or eating behaviors [17, 18, 19], data on weight changes during the transition to university are lacking. Available evidence indicates that Mexican university students exhibit a high prevalence of unhealthy eating behaviors [19], and overweight and obesity are more prevalent among men than women, a disparity likely linked to poor dietary habits and lifestyle choices [20]. Considering the increasing incidence and prevalence of obesity in Mexico, and the lack of regional information on the Freshman 15 phenomenon, the transition to college life may represent a strategic period for health promotion and the development of preventive interventions. Therefore, this study aimed to examine whether Mexican students gain weight during their first semester at university and to identify potential modifiable factors associated with this weight gain.

## 2 Material and Methods

### 2.1 Participants

**FIGURE 1:**
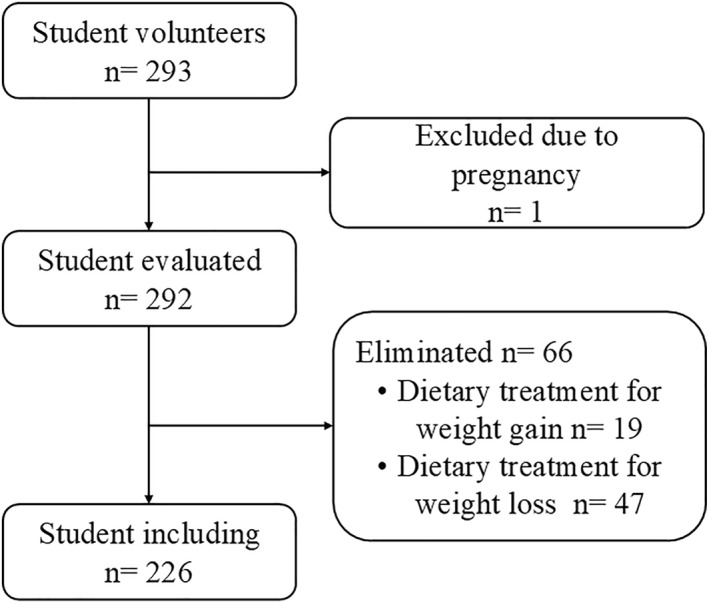
Flow chart with students excluded from the study.

This study utilized an observational pre‐post cohort design and assessed a convenience sample. The research was conducted in Culiacán Rosales, Sinaloa, Mexico. The sample was recruited through an open invitation to students who had recently enrolled in majors offered by a public university in northwestern Mexico (equivalent to the Freshman year in some English‐speaking countries). The study included students who met the following criteria: recent high school graduation, recent admission to a bachelor's degree program, ages between 17 and 21 years, and single without children. Women who became pregnant during the study period and those who initiated dietary treatment were eliminated (Figure 1). The evaluation period spanned from August to January of the school year, encompassing the first and last measurements.

### 2.2 Anthropometric Measurements

Trained research assistants, who had undergone standardized training before data collection, conducted all measurements during baseline and final assessments, following the methodology outlined by Gibson [21]. Participants were asked to remove their shoes, jackets, or sweaters, empty their pockets, and wear light clothing. Body weight, height, waist circumference, and hip circumference were measured using a digital scale (Tanita UM‐061, Illinois, USA), a stadiometer (Seca 214, Hamburg, Germany), and a flexible fiberglass tape with a scale ranging from 0 to 150 cm (Seca). Each measurement was taken twice, and the average value was recorded.

The classification of overweight and obesity was based on the World Health Organization (WHO) reference standards [22].

### 2.3 Lifestyle Habits Assessment

To collect information on the socioeconomic status of the participants, their eating habits, exercise habits and healthcare‐related behaviors, a survey proposed by Levitsky et al. 2004 [23] was translated into Spanish and adapted to the specific context of Mexican public universities, where structural and cultural factors, such as the absence of formal campus cafeterias, differ from the original setting. The survey encompassed various aspects such as feeding schedules, consumption of snacks, visits to regular or fast‐food restaurants, and alcohol and cigarette use. The Perceived Stress Scale (PSS), consisting of 14 items, was employed to evaluate participants' perceived stress levels. A numerical scale ranging from −3 (“much less/often”) to +3 (“much more/often”) was used to quantify responses. The scale aimed to assess the extent to which individuals appraised situations in their personal lives as stressful [24]. The surveys collected data retrospectively, covering a period of the past 6 months.

### 2.4 Statistical Analysis

The data collected in this study were analyzed using SPSS version 25. Descriptive statistics, including the mean and standard deviation, were calculated to summarize the distribution of continuous variables. Both paired and unpaired Student's t‐tests were used to compare means between the two groups. In cases where more than two groups were compared, analysis of variance (ANOVA) was applied. For analyzing categorical variables, the chi‐square test (*χ* ^2^) was utilized. Univariate and multiple linear regression analyses were performed to explore the relationship between changes in body weight and lifestyle characteristics. The model was adjusted for possible confounding variables such as overweight/obesity and exercise at baseline. Diagnostic collinearity was assessed using the variance inflation factor (VIF), yielding values ranging from 1.001 to 1.014, suggesting no evidence of collinearity [25]. *p* < 0.05 was considered statistically significant.

### 2.5 Ethics Approval

The study received approval from the Research Ethics Committee of the Faculty of Medicine at the Autonomous University of Sinaloa (registration number CONBIOÉTICA‐25‐CEI‐003‐20181012) before initiating the experiment and adhered to the principles outlined in the Helsinki Declaration. All participants provided informed consent to take part in the study.

## 3 Results

The sample consisted of 292 young individuals who met the criteria to participate in the study. However, 66 participants were excluded due to undergoing dietary treatment for weight gain or weight loss during the evaluation period. As a result, the final sample comprised 226 individuals, as indicated in Table 1.

**TABLE 1:** Final population distribution, by age and sex.

Age	Females (%)	Males (%)	All
17	5 (62.5)	3 (37.5)	8
18	115 (87.1)	17 (12.9)	132
19	44 (68.8)	20 (31.3)	64
20	3 (30.0)	7 (70.0)	10
21	6 (50.0)	6 (50.0)	12
All	173 (76.5%)	53 (23.5)	226

When analyzing changes in body weight regardless of sex, a statistically significant increase (*p* < 0.000) of 0.8 ± 2.2 kg was observed at the end of the first semester (60.9 ± 14.0 kg vs. 61.7 ± 14.3 kg). However, when considering sex, the increase was more pronounced, with a significant increase of 1.6 ± 2.6 kg for males and 0.5 ± 2.0 kg for females (Table 2). A significant increase in waist circumference was noted in women (1.1 ± 4.1 cm), while hip circumference increased significantly regardless of sex (1.4 ± 4.5 cm for males and 1.2 ± 5.6 cm for females). Based on the WHO cutoff points in Table 3, the initial weight status indicated that 23% of participants were classified as overweight or obese, similar to the national average for young adults. However, by the end of the study, there was a decrease in the percentage of individuals categorized as underweight or normal weight, accompanied by an increase of 3.5% in the overweight category. At the same time, in females, there was a more minor percentage increase for this category relative to males, at 3.5% and 3.8%, respectively (Table 3).

**TABLE 2:** Anthropometric changes during the first semester of undergraduate education.

Variable	Baseline (mean ± SD)	Final (mean ± SD)	Change	*p*
Weight (kg)				
Males (*n* = 53)	71.4 ± 18.6	73.0 ± 18.6	1.6 ± 2.6	**0.000**
Females (*n* = 173)	57.7 ± 10.4	58.4 ± 10.5	0.5 ± 2.0	**0.001**
All (*n* = 226)	60.9 ± 14.0	61.8 ± 14.3	0.8 ± 2.2	**0.000**
BMI (kg/m^2^)				
Males (*n* = 53)	24.0 ± 5.8	24.3 ± 5.7	0.3 ± 1.2	0.103
Females (*n* = 173)	22.5 ± 3.8	22.7 ± 3.9	0.3 ± 0.9	**0.000**
All (*n* = 226)	22.8 ± 4.4	23.1 ± 4.4	0.3 ± 1.0	**0.000**
Waist circumference (cm)				
Males (*n* = 53)	83.1 ± 18.3	82.6 ± 14.1	−0.5 ± 14.2	0.785
Females (*n* = 173)	71.7 ± 7.8	72.8 ± 8.3	1.1 ± 4.1	**0.001**
All (*n* = 226)	74.4 ± 12.2	75.1 ± 10.7	0.7 ± 7.7	0.191
Hip circumference (cm)				
Males (*n* = 53)	97.6 ± 11.3	99.0 ± 10.6	1.4 ± 4.5	**0.029**
Females (*n* = 173)	94.3 ± 8.1	95.5 ± 9.0	1.2 ± 5.6	**0.007**
All (*n* = 226)	95.1 ± 9.0	96.3 ± 9.5	1.2 ± 5.3	**0.001**

*Note:* The data represent the mean ± standard deviation (SD). Differences between means were analyzed by paired *t*‐test. Values in bold denote *p* < 0.05.

Abbreviation: BMI, body mass index.

**TABLE 3:** Classification of the population, according to body mass index.

Body mass index		Baseline (%)	Final (%)	*p*
Males (*n* = 53)	Underweight	7 (13.2)	7 (13.2)	0.911
	Normal	30 (56.6)	28 (52.8)	
	Overweight and obesity	16 (30.2)	18 (34)	
Females (*n* = 173)	Underweight	20 (11.6)	19 (11)	0.742
	Normal	117 (67.6)	112 (64.7)	
	Overweight and obesity	36 (20.8)	42 (24.3)	
All (*n* = 226)	Underweight	27 (11.9)	26 (11.5)	0.683
	Normal	147 (65.1)	140 (62)	
	Overweight and obesity	52 (23)	60 (26.5)	

*Note:* Frequency comparisons were performed by chi‐square analysis (*χ* ^2^).

Body weight changes were further analyzed according to various behavioral factors at the end of the semester. A significantly higher weight gain was noted in the male group compared with the female group (1.5 ± 2.6 and 0.5 ± 2.1 kg, *p* = 0.008). In most lifestyle cases, no significant differences were identified, except for the combination of residence type with household appliances (*p* = 0.003) and the time spent eating, which was marginally significant (*p* = 0.049) (Table 4). Individuals residing in a rooming house with fewer than four appliances experienced a significant average weight increase of 4.6 ± 4.5 kg compared with other categories. Additionally, participants who spent less than 20 min on meals showed a significant weight gain of 1.4 ± 2.2 kg compared with those who spent more than 20 min, gaining 0.6 ± 2.3 kg. No significant differences were observed in weight changes based on self‐perceived stress levels.

**TABLE 4:** Body weight changes according to sex and lifestyle.

Factor	Variable (*n*)	Body weight change (kg) (mean ± SD)	*p*
Sex	Males (53)	1.5 ± 2.6	**0.008**
	Females (173)	0.5 ± 2.1	
School shift	Morning (145)	0.9 ± 2.1	0.414
	Afternoon (81)	0.6 ± 2.4	
Address change	Yes (90)	1.0 ± 2.2	0.183
	No (131)	0.5 ± 2.3	
Type of address*	Living alone (29)	0.6 ± 2.5	0.313
	With family (186)	0.7 ± 2.1	
	Assisted home (6)	2.5 ± 3.8	
Exercise*	Stop (67)	0.5 ± 2.2	0.100
	Maintain (85)	0.8 ± 2.3	
	Never (59)	0.7 ± 2.3	
	Start (15)	2.1 ± 1.6	
Sleeping hours	Up 7 (29)	1. ± 1.9	0.573
	8 or more (197)	0.7 ± 2.3	
Consumption of soft drinks	Yes (176)	0.9 ± 2.1	0.420
	No (35)	0.4 ± 2.7	
Residence/possession of appliances*	Alone/up 4 (17)	−0.1 ± 2.3^a^	**0.012**
	Alone/5 or more (12)	1.7 ± 2.5	
	With family/up 4 (44)	1. ± 2.0	
	With family/5 or more (142)	0.6 ± 2.2^a^	
	Assisted home/up 4 (3)	4.6 ± 4.5^b^	
	Assisted home/5 or more (3)	0.3 ± 1.8	
Eating time	Up 20 min (44)	1.4 ± 2.2	**0.049**
	21 min or more (172)	0.6 ± 2.3	
Perceived stress scale (PSS)	Increased (101)	0.6 ± 2.3	0.202
	Decreased (94)	1.0 ± 2.2	

*Note:* The data represent the mean ± standard deviation (SD). Differences between means were analyzed by *t*‐test or *ANOVA. When ANOVA indicated significant differences, Tukey's post hoc test was performed to identify which groups differed. Different superscript letters denote statistically significant differences between groups. Values in bold denote *p* < 0.05.

The results of the univariate linear regression analysis identifying factors that influence body weight change are shown in Table 5. The regression model indicated that sex (female) (*β* = −0.929, *p* = 0.008) was significantly related to body weight change, while eating time (21 min or more) approached marginal significance (*β* = 0.756, *p* = 0.050). The normality of residuals was checked using a *Q*–*Q* plot and a histogram, both indicating an approximately normal distribution. Additionally, the Kolmogorov‐Smirnov test (*p* = 0.20) supported the model's validity.

**TABLE 5:** Univariate linear regression of lifestyle and body weight change.

Variables	β	SE	95% CI	t	*p*	Constant	*R* ^2^ adj
Sex	−0.929	0.348	−1.615, −0.242	−2.666	**0.008**	1.470	0.026
Eating time	0.756	0.383	0.002, 1.510	0.612	**0.050**	1.975	0.013
Address change	0.505	0.309	−0.105, 1.114	0.534	0.104	1.631	0.007
Times having breakfast at home	0.119	0.068	−0.015, 0.254	0.146	0.081	1.751	0.009

*Note:* Values in bold denote *p* < 0.05.

Additionally, we conducted a multiple linear regression analysis with body weight change as the dependent variable (Table 6). Based on the results of the univariate analyses, and although time spent eating barely reached significance, we decided to include it, along with sex and change of address, as independent variables. To control for type I error due to multiple testing, the Bonferroni correction was applied, adjusting the significance level to 0.0167 for model 1 and 0.01 for model 2. However, the sex variable showed a *p*‐value close to the corrected threshold (Model 1: *β* = −0.869, *p* = 0.018; Model 2: *β* = −0.945, *p* = 0.012), indicating that being female was associated with an average decrease in body weight change of approximately 0.9 and 1.0 kg, respectively. Additionally, no influence was observed for exercising or being overweight or obese. Since the adjusted *R* ^2^ values are low in both models (4.2%–4.4%), it suggests that, although the included predictors—mainly sex—have some influence, most of the variability in weight changes is not explained by the variables considered in the model.

**TABLE 6:** Multiple linear regression of lifestyle and body weight change.

Variable	Model 1						Model 2					
	*β*	SE	95% CI	*t*	*p*	VIF	*β*	SE	95% CI	*t*	*p*	VIF
Constant	1.062	0.357	0.358, 1.766	2.973	**0.003**		1.388	0.450	0.500, 2.275	3.083	**0.002**	
Sex	−0.869	0.365	−1.588, −0.150	−2.383	**0.018**	1.006	−0.945	0.372	−1.679, −0.211	−2.539	**0.012**	1.014
Eating time	0.706	0.385	−0.054, 1.465	1.639	0.068	1.007	0.713	0.389	−0.055, 1.481	1.832	0.068	1.011
Address change	0.517	0.316	−0.105, 1.139	1.832	0.103	1.001	0.568	0.321	−0.065, 1.202	1.769	0.078	1.002
Overweight/obesity							−0.165	0.377	−0.909, 0.578	−0.438	0.662	1.011
Exercise							−0.373	0.342	−1.047, 0.301	−1.091	0.276	1.001
	*F* = 4.120; *p* = 0.007; *R* ^2^ = 0.056; *R* ^2^ adj = 0.042						*F* = 2.921; *p* = 0.026; *R* ^2^ = 0.067; *R* ^2^ adj = 0.044					

*Note:* Model 1 corresponds to the unadjusted analysis, whereas model 2 was adjusted for potential confounding variables, including overweight/obesity and exercise. Values in bold denote *p* < 0.05.

Abbreviations: CI, confidence interval; SE, standard error; VIF, variance inflation factors.

## 4 Discussion

The present study aimed to investigate weight gain among students at a public university in northwestern Mexico during their first semester and the associated modifiable factors. The main finding revealed an average increase of 0.8 ± 2.2 kg during the first semester, comparable to other universities worldwide, where weight gains ranging from 0.7 to 4 kg have been observed [26, 27, 28, 29, 30, 31, 32]. In this study, weight change varied considerably, ranging from −8.1 to +9.1 kg overall (males: −6.1 to +9.1 kg; females: −8.1 to +7.9 kg). Other studies have reported even broader ranges, including −5.5 to +11.8 kg [30], −8.7 to +16.8 kg [5] and −13.2 to +20.9 kg [33]. These differences may be associated to the length of the observation period, as some studies have reported weight gains exceeding 5 kg and up to 15 kg over the course of a full academic year [7, 34, 35, 36]. Although the average weight gain of 0.8 kg over 6 months may appear modest and of limited immediate clinical impact, it was statistically significant and corresponds to an approximate additional energy intake of 30 kcal/day. This finding underscores that weight increases during adolescence occur gradually and may not be visibly apparent. However, even small increments, when sustained over time, can contribute meaningfully to the development of overweight and obesity in young adulthood and have significant health consequences [23]. Moreover, high weight variability in young women may indicate a breakdown in body weight regulatory systems and predict long‐term weight gain [37].

Analyzing the modifiable factors associated with weight change can be challenging due to the diverse circumstances that individuals experience, making direct comparisons between studies difficult. As the school semester progresses, evidence shows an increase in the consumption of unhealthy snacks, and other studies confirm a decline in diet quality during the transition to adulthood [38, 39]. It has been observed that food conscientiousness serves as a significant psychological factor influencing weight gain among university students. In this regard, those with a high level of food conscientiousness (+1 SD) experience a lower weight increase (0.1 kg) compared to their peers with a low level of food awareness (−1 SD), who, on average, gain 1.5 kg [29]. Likewise, a strong hedonic attraction to palatable foods is suggested to be a potential risk factor for both the maintenance and onset of loss of control eating [40].

On the other hand, leaving the parental home and assuming responsibility for food provision often negatively impacts eating habits [41, 42]. In addition, greater weight gain has been reported among individuals living in residences compared to those living at home [31]. Although this study population did not include students living on campus, a similar situation, such as residing in a rooming house, also negatively influenced students' eating habits. This situation and limited access to kitchen appliances may lead to a greater reliance on food from outside sources. However, it is important to acknowledge that the present study had limitations as we lacked data to analyze diet and draw specific inferences directly. Instead, we focused on examining circumstances indirectly associated with eating habits.

Previous studies conducted with adolescents have consistently shown a decline in physical activity levels during the transition from school to young adulthood [43, 44, 45, 46]. A significant decrease in the daily frequency of physical activity, approximately 6% per semester, has been observed from the first to the seventh semester [47]. A meta‐analysis of physical activity patterns among university students estimates that between 40% and 50% of this population is physically inactive, and that interventions implemented to date have yielded only moderate improvements in physical activity levels [48]. In the present work, 69.3% of undergraduates reported engaging in exercise during the initial assessment; however, only 44.2% maintained or initiated exercise by the end of the first semester. Interestingly, the highest weight gain was observed in those who started exercising; however, this behavior could not be attributed to a cause‐and‐effect relationship. It is possible that those who gained weight sought ways to counteract it.

Prior research has indicated that factors such as access to unhealthy foods, nighttime snacking, alcohol consumption, eating due to stress or boredom, and easy access to food in one's living environment are all associated with weight gain [49, 50, 51]. The primary purpose of this study was to raise awareness among young individuals about their potential vulnerability to weight gain, as this knowledge can influence their behavior and encourage the adoption of healthy habits. Social support can serve as a protective factor by mitigating the impact of stress‐related eating on weight gain during the freshman year of college. By promoting social support and emphasizing the importance of healthy behaviors, interventions can be designed to help young people navigate this critical period and prevent excessive weight gain [52].

Starting university with stronger self‐regulation skills related to eating may help students maintain or achieve a healthy diet, potentially shielding them against significant weight gain, especially among those who are already overweight [9]. Future research should explore the intensity of physical activity or incorporate dietary interventions and lifestyle changes to prevent weight gain during the first year of college [27, 53]. Although this work was not conducted in an urban or rural setting, we agree that, in addition to known risk factors, the local environment and cultural practices may contribute to the prevalence of overweight and obesity [54]. Researchers, health authorities, and other stakeholders should consider the unique characteristics of public universities without dining halls or student housing when developing effective health policies.

Although the current research study did not find a relationship between PSS and weight gain, this results align with those of another study [30]. However, perceived stress is suggested to be a potential causal factor in weight fluctuations, particularly among female university students [55]. It is possible that stressed students engage in less physical activity, eat more, and choose less healthy options; however, the effects of these changes may not have been directly reflected in weight gain at the time of measurement [30]. Furthermore, it has been observed that higher perceived stress and lower coping resources may contribute to emotional eating during the transition to university [56]. University students who experience high levels of perceived stress tend to adopt more unhealthy eating habits, such as frequently consuming ready‐made meals [57]. This increase in stress not only negatively affects their food choices but can also lead to health problems such as obesity [58].

The results conducted align with those reported in other studies, showing that sex is an influential predictor of weight gain, with males exhibiting more adverse changes compared to female university students [33, 35, 59, 60]. This may be attributed to the lower level of concern about body weight observed among male students and their use of fewer strategies to prevent weight gain compared to female [61]. Additionally, male gender is a risk factor for overweight and obesity (OR = 1.8) [62]. They also found that certain dietary habits were associated with weight gain, such as consuming fried foods 7 days a week (OR = 88.3), nightly snacks 7 days a week (OR = 13.2), and dietary preferences for sweet (OR = 26.3), salty (OR = 14.7), and spicy (OR = 1.9) foods [62]. Conversely, a large retrospective cohort study indicated that skipping dinner is a significant predictor of weight gain and overweight/obesity in both male and female students [63]. In contrast, in female first‐year university students, weight suppression has been identified as an independent predictor of fat gain over a 2‐year period (OR = 1.87) [14].

The assessment of neural vulnerability factors has shown that increased activation of brain regions involved in reward and incentive valuation, particularly in response to food‐related cues, anticipation, and consumption of high‐calorie foods, is linked to a higher risk of future weight gain and poorer results in behavioral weight loss treatments. These findings highlight the need for further research into neural vulnerability factors that may increase the risk of developing obesity [64].

This study has several limitations that should be acknowledged. First, the use of a convenience sample limits the generalizability of the findings to broader populations, particularly beyond the specific geographic and demographic characteristics of the participants. Additionally, no formal sample size calculation was conducted; however, all students who met the eligibility criteria and agreed to participate within the enrollment period were included. Second, although the study used validated tools for lifestyle assessments, the survey was adapted to the Mexican public university context to preserve the content and intent of the original items and was pre‐tested for clarity. Still, no formal validation or reliability analysis was conducted for the adapted version. Third, the observational pre‐post design without a control group precludes establishing causality and limits the ability to disentangle the influence of time‐related confounders. Fourth, although the average weight gain observed was statistically significant, the magnitude of this change (0.8 kg) may be considered modest and of limited immediate clinical relevance. Nonetheless, it should be interpreted as an early signal of gradual and potentially cumulative weight increases during late adolescence, a period known to set trajectories for overweight and obesity in adulthood. Finally, the relatively low explanatory power of the regression models suggests that other unmeasured factors, such as dietary intake, physical activity intensity, or psychological determinants, may play a more significant role in body weight changes during the transition to university life.

In conclusion, the findings presented demonstrate a significant increase in body weight among young individuals recently enrolled in a public university, highlighting this period as potentially vulnerable. Male gender proved to be a predictor of weight gain, making it crucial to gather information that characterizes the habits and behaviors during this life stage, tailored to each gender, to promote healthier eating habits during the transition to university life. Therefore, further studies on specific behaviors and health habits are needed to accurately identify the modifiable factors associated with weight gain.

## Author Contributions

Study design: Javier Abednego Magaña Gómez, Gisela Duarte de la Peña, Yuridia Chaidez Fernández, and Wendy Paola Gastélum Espinoza. Data collection: Javier Abednego Magaña Gómez, Loranda Calderón Zamora, Yuridia Chaidez Fernández, Dalia Magaña Ordorica, Wendy Paola Gastélum Espinoza, and Francisco Castro Apodaca. Data analysis and interpretation: Javier Abednego Magaña Gómez, Alma Marlene Guadrón Llanos, Francisco Castro Apodaca, and Wendy Paola Gastélum Espinoza. Manuscript writing: Wendy Paola Gastélum Espinoza, Alma Marlene Guadrón Llanos, Gisela Duarte de la Peña, Loranda Calderón Zamora, Francisco Castro Apodaca, Dalia Magaña Ordorica, Yuridia Chaidez Fernández, and Javier Abednego Magaña Gómez.

## Funding

Programa de Fomento y Apoyo a Proyectos de Investigación (Profapi) from the Universidad Autónoma de Sinaloa, academic grant PROFAPI2011/99.

## Conflicts of Interest

The authors declare no conflicts of interest.
